# 25(OH)-Vitamin D alleviates neonatal infectious pneumonia via regulating TGFβ-mediated nuclear translocation mechanism of YAP/TAZ

**DOI:** 10.1080/21655979.2021.1990000

**Published:** 2021-10-27

**Authors:** Qi Sun, Yiwen Gao, Lina Qiao, Yi Yuan, Qian Liu

**Affiliations:** a Department of Pediatric, Affiliated Hospital of Weifang Medical University, Weifang, Shandong, China; b Institute of Life Sciences, Jiangsu University, Zhenjiang, Jiangsu, China

**Keywords:** Neonatal infectious pneumonia (nip), 25-oh-vd, inflammatory indicators, deficiency, yap/taz, anti-inflammatory mechanism

## Abstract

Neonatal infectious pneumonia (NIP) is a common infectious disease that develops in the neonatal period. The purpose of our study was to explore the potential roles of 25(OH)-Vitamin D (25-OH-VD) and its anti-inflammatory mechanism in NIP. The results showed that serum 25-OH-VD level was negatively correlated with the severity of NIP, whereas Spearman’s correlation analysis showed a significant positive correlation between the severity of NIP and the levels of pneumonia markers procalcitonin (PCT) and interleukin-6 (IL-6). The expression of vitamin D receptor (VDR) was down-regulated, while the transforming growth factor β (TGFβ), nuclear YAP, and TAZ were up-regulated in the peripheral blood mononuclear cells (PBMCs) of neonates with severe pneumonia. Neonates with 25-OH-VD deficiency were associated with an increased risk of NIP. In BEAS-2B cells, down-regulation of nuclear YAP and TAZ was found in the lipopolysaccharide (LPS) + VD group relative to the LPS-induced group. Additionally, positive rate of nuclear YAP, as detected by immunocytochemistry (ICC), and the nuclear translocation of nuclear YAP/TAZ by IFA in the LPS+VD group showed an intermediate level between that of the control and LPS-induced groups. Furthermore, the expressions of VDR and CYP27B1 were significantly increased in the LPS+VD group as compared to those in the LPS-induced group. The anti-inflammatory mechanism in NIP was achieved due to the 25-OH-VD mediating TGFβ/YAP/TAZ pathway, which suggested that using 25-OH-VD might be a potential strategy for NIP treatment.

## Introduction

Neonatal pneumonia, particularly severe neonatal infectious pneumonia (NIP), is the most common infectious disease among neonates and a crucial cause of neonatal mortality [1]. Common therapeutics for NIP include intravenous glucocorticoids [2], antibiotics [3], and intravenous immunoglobulins (IVIG) [4]. However, the side effects of these therapies cannot be ignored. For instance, long-term glucocorticoid therapeutics affect innate and acquired immunity, thus increasing the risk of infection [5]. Antibiotics lead to drug dependence and resistance [6], while IVIG treatment leads to adverse effects such as hypertension and anaphylaxis [7]. It has been reported that vitamin D deficiency was associated with community-acquired pneumonia [8] and COVID-19 infectious pneumonia [9], while there has been little research on NIP.

Vitamin D is a steroid hormone originating from cholesterol. It is an essential component of human health and is involved in several metabolic and immunological processes [10,11]. The deficiency of vitamin D has become a global health issue and has been found to be associated with several pathological conditions [10,12].

Vitamin D can be produced by most plants and animals that are exposed to sunlight [13]. *In vivo*, vitamin D can be activated through a two-step hydroxylation process. First, vitamin D is converted to its major circulating form, 25-hydroxy vitamin D (25-OH-VD), under the action of P450 enzymes such as CYP2R1 and CYP27A1 (25-hydroxylases) in the liver. Then, 25-OH-VD is converted to 1,25-hydroxyvitamin D (1,25-OH-2D) in the kidneys under the action of CYP27B1 (1α,25-hydroxylase) [10,13,14]. 1,25-OH-2D is finally recognized and bound to the vitamin D receptor (VDR) in the target cells [15–17]. The half-life of 25-OH-VD is more stable than 1,25-OH-2D and the serum level is higher and can be easily detected. Thus, 25-OH-VD has become a reliable indicator of individual vitamin D levels [10,14]. The VDR is a nuclear receptor that presents a part of homodimers of VDR or heterodimers of VDR/RXR to target cell DNA and regulates the synthesis of special proteins [18,19]. VDR plays a role in suppressing transforming growth factor β (TGFβ)-SMAD signal transduction in the murine renal epithelial TCMK-1 cell line [20]. The activation of TGF-β signaling undermines the defense system of the host, leading to an aggravated bacterial infection [21]. More importantly, TGFβ can facilitate YAP activation and nuclear translocation [22]. The active YAP increases during lung inflammation [23], and is positively related to the secretion of pro-inflammatory factors (including IL-2, IFN-γ, TNFα, and IL-1β) in liver inflammation [24]. These characteristics are consistent with their changes in infectious pneumonia. Nevertheless, the potential mechanism of whether vitamin D exerts anti-inflammatory effect via VDR or YAP/TAZ has not been explored. Infectious pneumonia is a common lung disease caused by infection of bacteria, viruses, or fungi [25,26]. The severity levels of pneumonia are correlated with the disease-resistant and tissue recovery mechanisms of patients [27].

As a major bioactive component of the cell wall, lipopolysaccharide (LPS) is an endotoxin produced by Gram-negative bacteria [28]. LPS has also been reported to act as a risk factor for the induction of inflammation associated with pneumonia [29–31]. In this study, we used LPS-induced BEAS-2B to mimic a cell model of infectious pneumonia and explored the mechanism of action of 25-OH-VD on this model.

In this study, we concentrated on investigating the relationship between vitamin D deficiency and NIP. Blood samples were collected from pneumonia patients and healthy controls. Then, the expressions of 25-OH-VD, VDR, TGFβ, IL-2, IFN-γ, TNFα, IL-1β, Ca^2+^, PCT, CRP, and IL-6 in mild pneumonia, severe pneumonia, and healthy controls were determined using qRT-PCR, western blot, ELISA, and calcium colorimetry assay. After constructing an in vitro pneumonia model by exposing BEAS-2B cells to LPS, we further determined the effects of VD treatment on LPS-triggered BEAS-2B cell proliferation, apoptosis, and YAP/TAZ activation.

## Materials and methods

### Blood collection from the neonates

The basic information for different infectious levels of neonates was collected from the Department of Pediatric in Affiliated Hospital of Weifang Medical University. A total of 65 neonates with infectious pneumonia were divided into two groups: 34 mild cases and 31 severe cases. In addition, 60 healthy neonates were chosen as the control group. The basic information of neonates was collected from the medical record system. The basic information of the three groups (severe cases, mild cases, and control) were compared proportionally. The venous blood of the neonates was collected within 24 h after diagnosis. All participants’ guardians had provided informed consents for inclusion in the study.

### Enzyme-linked immunosorbent assay (ELISA)

The ELISA assay was conducted as described before [32]. The levels of 25-OH-VD and neonatal pneumonia indicators including procalcitonin (PCT), c-reaction protein (CRP), and interleukin-6 (IL-6) were measured in the volunteers’ serum using an ELISA kit (Sigma, USA). In addition, the levels of inflammatory markers in plasma, including IL-2, IFN-γ, TNF-α, and IL-1β, were also measured using ELISA (Sigma, USA).

### Calcium colorimetry assay

A Calcium Detection Assay kit (ab102505, Abcam, UK) was used for detecting Ca^2+^ in serum [33]. Briefly, a standard curve was obtained by using a fresh set of Ca^2+^ standard solutions. Then, the optical density of the blood sample was measured at 575 nm. Finally, the level of Ca^2+^ was obtained by matching the optical density of the sample through the standard curve, which was then multiplied by the dilution ratio to quantify the level of blood Ca^2+^.

### Cells culture and treatment

The human lung epithelial cells (BEAS-2B) were inoculated in DMEM, which had a high glucose content, purchased from Lonza BioWhittaker^TM^ with 10% fetal bovine serum (FBS, Sigma, USA). A 0.25% EDTA-Trypsin solution was used to digest the cells at a logarithmic growth phase. The digested cells were re-suspended in fresh DMEM (high glucose) with 10% FBS and transferred into separate culture dishes for further use. The LPS-induced inflammation group was inflamed by the addition of 0.5 µg/ml LPS (LPS diluted with saline) and cultured for 24 h at room temperature and 5% CO_2_. After 24 h of cultivation, the LPS-induced inflammation group was further inflamed by addition of 100 nmol/l 25-OH-VD (LPS+VD, 25-OH-VD dissolved in 100% ethanol) to prepare the LPS+VD administration group [34].

### Cell counting kit-8 assay (CCK-8)

The CCK8 assay was performed as described before [35] to determine the cell viability among the control, LPS, and LPS+VD groups. The BEAS-2B cells (2 × 10^5^/well) were first seeded in 96-well plates for 0, 6, 12, 24, and 48 h, followed by incubation with CCK8 reagent for 2 h at 37°C. The absorbance was read under a BioTek plate reader (SynergyH4, USA) at 450 nm. The cell viability of the LPS and LPS+VD groups was calculated and compared with that of the control.

### Quantitative real-time PCR (qRT-PCR)

qRT-PCR was used to evaluate the mRNA expression levels of VDR and CYP27B1 in BEAS-2B cells, as well as the expression levels of VDR and TGFβ in PBMCs. First, total RNA was extracted using the Trizol kit (Invitrogen, USA) and reverse-transcribed into cDNA using a first-strand synthesis kit (Thermo, USA). Then, qRT-PCR were conducted with Taq DNA polymerase (Takara, Japan). The primer sequences of CYP27B1, VDR, TGFβ, and the reference gene GAPDH, listed in Table 1, were synthesized by Invitrogen (Thermo, USA). StepOne qPCR (ABI, USA) was used to detect and the 2^−ΔΔCt^ method was used for the analysis of relative mRNA expression levels.

**Table 1.** The sequences of VDR, CYP27B1, TGFβ and GAPDH

Primer name	Primer sequences
VDR	Forward: 5-TGCCTGACCCTGGAGACTTTGACC-3 Reverse: 5-CATCATGCCGATGTCCACACAGCG-3’
CYP27B1	Forward: 5-TGGCCCAGATCCTAACACATTT-3 Reverse: 5-GTCCGGGTCTTGGGTCTAACT-3
TGFβ	Forward: 5-TACCTGAACCCGTGTTGCTCTC-3 Reverse: 5-GTTGCTGAGGTATCGCCAGGAA-3
GAPDH (Reference)	Forward: 5-GGTATCGTGGAAGGACTCATGAC-3 Reverse: 5-ATGCCAGTGAGCTTCCCGTTCAGC-3

### Western blot assay

First, the nuclear/cytoplasmic fractionation of cells was conducted as described before [36]. Nuclei extracts were collected by centrifugation at 4°C from the supernatant after a series of washing and lysing. Then, the expression levels of VDR, TGFβ, nuclear YAP and TAZ (n-YAP and n-TAZ), IL-2, IFN-γ, TNF-α, and IL-1β were detected using the western blot assay with β-actin as internal reference. The primary and secondary antibodies used were all rabbit anti-human antibodies (Invitrogen, USA): anti-VDR (1:1000), anti-TGFβ (1:2000), anti-n-YAP (1:1000), anti-n-TAZ (1:5000), anti-IL-2 (1:1000), anti-IFN-γ (1:1000), anti-TNFα (1:1000), anti-IL-1β (1:1000), β-actin (1:1000), and goat anti-rabbit IgG H&L (DyLight®488) preadsorbed (1:1000). The gray value of the target protein was calculated using ImageJ, the error was corrected with the internal parameter gray value, and the relative expression levels of the target protein were also calculated.

### Immunocytochemistry

ICC was applied to detect the nuclear translocation of YAP in each 48 h treated group [37]. PBS solution was set as the negative control. The positive expression of YAP displayed brownish yellow granules, which were found in both cytosol and nucleus. The nuclear positive cells in each of the 10 randomly selected high-power fields were counted under a microscope. The nuclear positive rate of YAP was then calculated.

### Immunofluorescence assay (IFA)

Similarly, IFA was applied to detect the nuclear translocation of YAP/TAZ complex in each 48 h treated group [38]. The BEAS-2B cells were first fixed, penetrated, and blocked, followed by the addition of primary antibody anti-YAP/TAZ (Cell Signaling, USA, 1:50). The goat anti-rabbit IgG antibody (Invitrogen, USA) was then added and incubated at room temperature for 45 min. DAPI (2 mg/ml) was applied for nucleus staining and images were taken by a fluorescent inverted microscope (Ti2-U, Nikon, Japan).

### Statistical analysis

Spearman rank correlation analysis was used to analyze the correlation between serum level of 25-OH-VD and the indicators of NIP. All data were shown as the mean ± standard deviation. The difference of continuous variables among groups was appraised using either a *t*-test or one-way ANOVA. Values of *p* < 0.05 were regarded as statistically significant.

## Results

In this study, we investigated the serum level of 25-OH-VD, Ca^2+^, PCT, CRP, IL-6, VDR, TGFβ, and inflammatory cytokines in NIP patients. The correlation between aberrant expression of 25-OH-VD and the severity of NIP was subsequently analyzed. Additionally, the anti-inflammatory mechanism of vitamin D was also investigated. Finally, the inhibitory effects of VD treatment on nuclear translocation of YAP/TAZ complex was verified.

### The relationship between 25-OH-VD deficiency and NIP

The basic information of the neonates is proportionally listed and compared in Table 2. As shown in Table 2, there was no significant difference in the basic information among the three groups. This indicated that the results of the following experiments were not affected by gender, gestational age, birth weight, delivery mode, season of birth, and maternal illness. In order to further verify the relationship between 25-OH-VD and NIP, the serum levels of 25-OH-VD among three groups were detected using ELISA and the deficiency rate of 25-OH-VD was calculated, as shown in Table 3. The serum 25-OH-VD deficiency rates of pneumonia patients (both mild and severe cases) were higher than that of the control group. In particular, the percentages of deficient and seriously deficient cases in the severe group (64.52% and 29.03%, respectively) were higher relative to those in the mild group (64.71% and 11.76%, respectively). However, severe deficiency of 25-OH-VD was not observed in the control group and the normal cases that existed in the severe pneumonia group were found. On the contrary, the normal and insufficient rates of 25-OH-VD in severe infectious pneumonia cases were reduced (0 and 6.45%, respectively). These results indicated that severe deficiency of 25-OH-VD was a characteristic of severe pneumonia.

**Table 2.** Basic information in the control, mild infectious pneumonia and severe infectious pneumonia cases

**Group**	**Cases**	**Gender (male/female)**	**Gestational age** **(x ± s, week)**		**Birth weight** **(x ± s, g)**	**Delivery mode (n (%))**		
						**Cesarean delivery**		**Normal delivery**
**Control**	60	29/31	38.2 ± 0.7		2963 ± 230	31 (51.7)		29 (48.3)
**Mild pneumonia**	34	16/18	38.2 ± 0.9		2861 ± 284	16 (47.1)		18 (52.9)
**Severe pneumonia**	31	15/16	38.3 ± 1.1		2863 ± 304	17 (54.8)		14 (45.2)
***F* value**		0.0707	0.3012		2.356	0.1972		
***p* value**		0.9318	0.7404		0.0991	0.8213		
**Group**	**Season of birth (n (%))**				**Maternal illness during pregnancy (n (%))**			
	**Spring**	**Summer**	**Autumn**	**Winter**	**Infection**	**Diabetes**	**Hypertension**	**Thyroid hypo-function**
**Control**	13 (21.7)	15 (25.0)	14 (23.3)	18 (30.0)	0	10 (16.7)	8 (13.3)	5 (8.3)
**Mild pneumonia**	8 (23.5)	7 (20.6)	10 (29.4)	9 (26.5)	1 (2.9)	3 (8.8)	4 (11.8)	5 (14.7)
**Severe pneumonia**	6 (19.3)	8 (25.8)	7 (22.6)	10 (32.3)	2 (6.5)	4 (12.9)	7 (22.6)	3 (9.7)
**F value**	0.0525				0.5939			
***p* value**	0.9489				0.5538			

*p* > 0.05 was performed to eliminate the effects caused by gender, gestational age, birth weight, delivery mode, season of birth and the maternal hypertension during pregnancy etc. on the results of 25-OH-VD.

**Table 3.** Comparison of 25-OH-VD deficiency rate among the controls, mild infectious pneumonia and severe infectious pneumonia cases

Group	Cases	Normal (n (%))	Insufficient (n (%))	Deficient (n (%))	Seriously deficient (n (%))
Control	60	12 (20.00)	27 (45.00)	21 (35.00)	0
Mild pneumonia	34	2 (5.88)	6 (17.65)	22 (64.71)	4 (11.76)
Severe pneumonia	31	0 (0.0)	2 (6.45)	20 (64.52)	9 (29.03)

According to the ‘Prevention and control proposal of the deficiency of micronutrients in children’ (prevention and control proposal), serum 25-OH-VD > 20.0 µg/L was considered as the normal level, 15.0 ~ 20.0 µg/L as the insufficient level, 5.1 ~ 14.9 µg/L as the deficient level, and ≤ 5.0 µg/L as the severely deficient level. In this study, serum 25-OH-VD ≤ 15.0 µg/L data were collected and calculated the rate of deficiency.

### Correlation between the levels of 25-OH-VD and Ca^2+^, PCT, CRP, and IL-6

**Figure 1.**
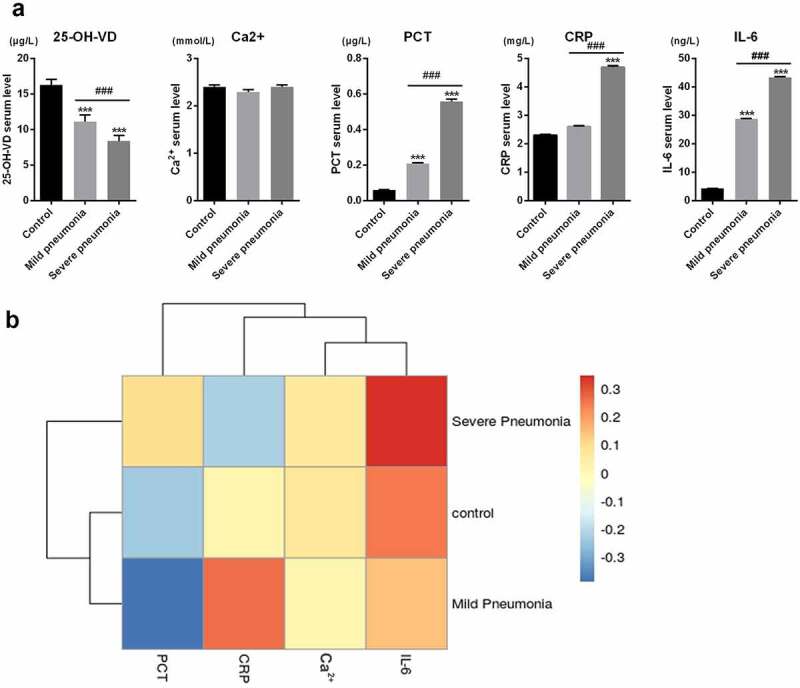
**The correlation between the levels of 25-OH-VD and Ca^2+^, PCT, CRP, and IL-6**. (a) Statistical analysis of serum levels of 25-OH-VD, Ca^2+^, PCT, CRP, and IL-6 in the controls and mild and severe infectious pneumonia cases. (b) Correlations between the 25-OH-VD levels and four inflammatory indicators. ‘***’ *p* < 0.001 vs. control; ‘^###^’ *p* < 0.001 vs. mild pneumonia cases

In NIP, serum Ca^2+^, PCT, CRP, and IL-6 are considered as inflammatory indicators. The changes in serum levels of these inflammatory indicators among three groups were statistically analyzed, and the results are presented in Table 4 and Figure 1a. There was no significant difference in the serum level of Ca^2+^ among the control, mild, and severe pneumonia cases. However, significant increase in CRP was found in severe pneumonia cases compared with those in control cases. In addition, prominent increases in PCT and IL-6 levels were observed in mild and severe pneumonia cases as compared to those in control cases. In addition, there were significant differences in PCT, CRP, and IL-6 levels between severe and mild pneumonia cases. Unlike the above-mentioned indicators, the serum level of 25-OH-VD was lower in neonatal pneumonia patients, particularly in severe cases (*p < *0.001). This indicated that the level of serum 25-OH-VD was negatively correlated with the levels of NIP inflammatory indicators.

**Table 4.** Statistical analysis of serum levels of 25-OH-VD, Ca^2+^, PCT, CRP and IL-6 in the controls, mild infectious pneumonia and severe infectious pneumonia cases

Group	Cases (n)	25-OH-VD (µg/L)	Ca^2+^ (mmol/L)	PCT (µg/L)	CRP (mg/L)	IL-6 (ng/L)
Control	60	16.28 ± 0.770	2.405 ± 0.042	0.060 ± 0.003	2.304 ± 0.032	4.305 ± 0.051
Mild pneumonia	34	11.13 ± 0.939	2.300 ± 0.048	0.210 ± 0.005	2.601 ± 0.040	28.80 ± 0.196
Severe pneumonia	31	8.433 ± 0.773	2.403 ± 0.046	0.559 ± 0.014	4.700 ± 0.048	43.40 ± 0.247
*F* value		23.63	1.536	1304	1011	19,278
*p* value		< 0.001	0.2195	< 0.001	< 0.001	< 0.001

*p* < 0.05 was considered to indicate a statistically significant result. The statistical differences were found in the serum levels of 25-OH-VD, PCT, CRP and IL-6 among the controls, mild pneumonia cases and severe pneumonia cases but not Ca^2+^.

The correlation between 25-OH-VD and levels of NIP indicators (including Ca^2+^, PCT, CRP, and IL-6) is shown in Table 5. As shown in Table 5 and Figure 1b, Spearman rank correlation analysis was used to analyze the relationship between serum level of 25-OH-VD and the levels of indicators of NIP. There was a negative correlation between the serum levels of PCT and IL-6 and the serum level of 25-OH-VD, with *r* values of – 0.340 and – 0.345, respectively (*p < *0.001). No significant correlation was found between the levels of Ca^2+^, CRP, and 25-OH-VD.

**Table 5.** Correlations of 25-OH-VD with Ca^2+^, PCT, CRP and IL-6 among the neonates with infectious pneumonia

Indicators	r_s_ value (95%CI)	*p* value
Ca^2+^	0.042 (−0.121,0.210)	0.636
PCT	−0.340 (−0.463, −0.184)	<0.001
CRP	−0.135 (−0.298, 0.027)	0.113
IL-6	−0.345 (−0.481, −0.170)	<0.001

*p* < 0.05 was considered to indicate a statistically significant result. 25-OH-VD negatively correlate to serum levels of PCT and IL-6. 25-OH-VD did not correlate to serum levels of Ca^2+^ and CRP.

### The anti-inflammatory mechanism of 25-OH-VD in NIP

**Figure 2.**
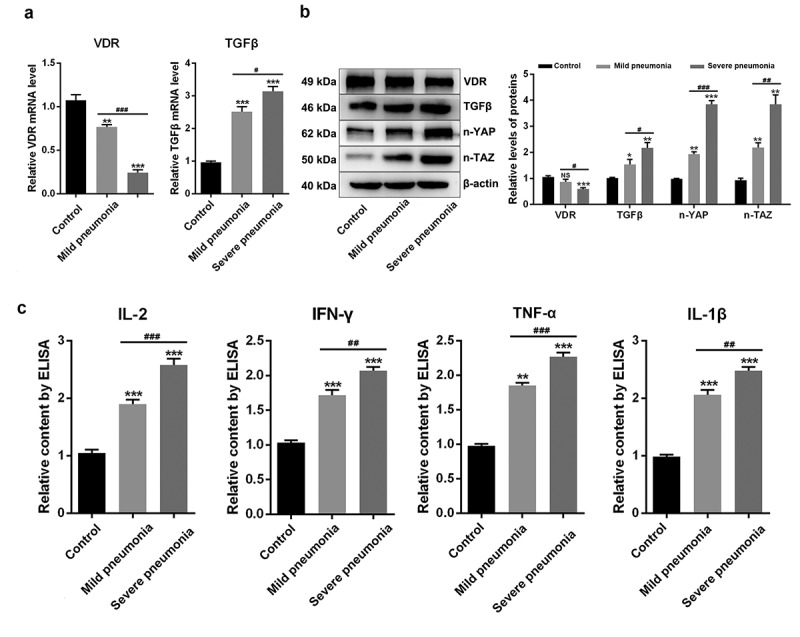
**The analysis of TGFβ/YAP/TAZ pathway-related genes**. (a) The mRNA expression levels of VDR and TGFβ in PBMCs, as determined using qRT-PCR. (b) Protein expression levels of VDR, TGFβ, n-YAP, and n-TAZ, as obtained using the western blot method. (c) Levels of inflammatory indicators IL-2, IFN-γ, TNFα and IL-1β, as determined using ELISA. NS, no significant difference with control; ‘*’ *p* < 0.05, ‘**’ *p* < 0.01, and ‘***’ *p* < 0.001 vs. control; ‘^#^’ *p* < 0.05, ‘^##^’ *p* < 0.01, and ‘^###^’ *p* < 0.001 vs. mild pneumonia cases

To explore the relationship between inflammation and NIP, the expressions of TGFβ/YAP/TAZ pathway proteins and downstream inflammatory indicators were detected. First, the expression levels of VDR and TGFβ in PBMCs were detected using qRT-PCR, as shown in Figure 2a. The level of VDR in PBMCs was slightly down-regulated in mild cases and significantly down-regulated in severe cases as compared to those in the controls (*p < *0.01). However, the level of TGFβ was extremely high in mild cases and was continuously raised in severe cases as compared to that in the controls (*p < *0.001).

To study the role of TGFβ/YAP/TAZ in the inflammatory mechanism of NIP, the expression levels of VDR, TGFβ, n-YAP, and n-TAZ in PBMCs were detected using the western blot method, and the results are presented in Figure 2b. As depicted in Figure 2b, the expression levels of TGFβ, n-YAP, and n-TAZ in PBMCs were significantly up-regulated, while that of VDR was down-regulated with the increase in the severity of cases (*p < *0.01). Then, the levels of inflammatory indicators were detected using ELISA. As shown in Figure 2c, the levels of inflammatory indicators IL-2, IFN-γ, TNF-α, and IL-1β increased gradually with the increase in the severity of the cases (*p < *0.01). These findings revealed that YAP and TAZ were highly active and the levels of these inflammatory indicators were up-regulated in severe NIP cases.

### VD treatment inhibited the nuclear translocation of YAP/TAZ complex in BEAS-2B cells

**Figure 3.**
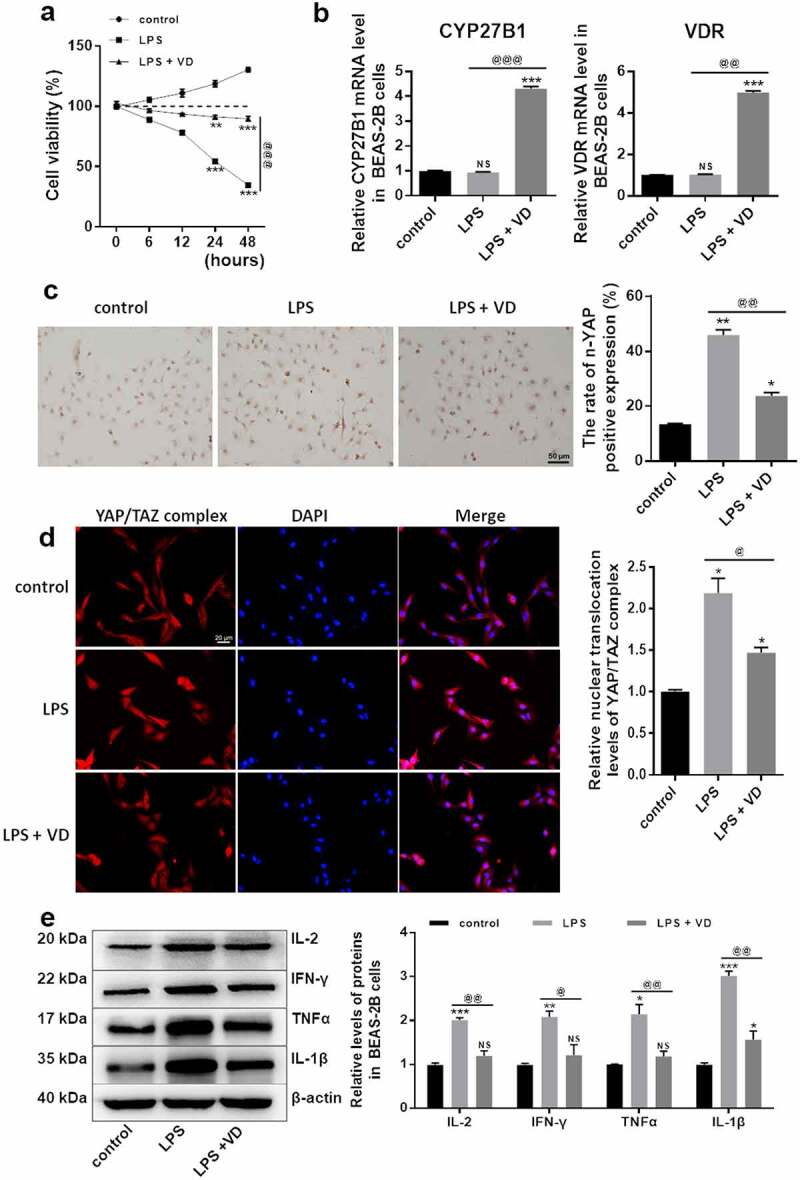
**25-OH-VD inhibited the nuclear translocation of YAP/TAZ complex**. (a) Cellular viability of BEAS-2B cells, as determined using CCK8 assay. (b) mRNA expression levels of CYP27B1, VDR, and TGFβ, as detected using qRT-PCR. (c) The positive expression of YAP in nuclei, as detected using ICC and concluded in a histogram; Bar: 50 μm. (d) IFA was applied to confirm the nuclear translocation of YAP/TAZ complex in BEAS-2B cells; Bar: 20 µm. (e) Expression levels of IL-2, IFN-γ, TNF-α, and IL-1β, as determined using the western blot method. NS, no significant difference with control; ‘*’ *p* < 0.05, ‘**’ *p* < 0.01, and ‘***’ *p* < 0.001 vs. control; ‘^@^’ *p* < 0.05, ‘^@@^’ *p* < 0.01, and ‘^@@@^’ *p* < 0.001 vs. LPS group

The cell viability of cultured control, LPS, and LPS+VD treated BEAS-2B cells at 0, 6, 12, 24, and 48 h was detected using CCK8 assay. As shown in Figure 3a, the viability of cells with LPS treatment was significantly decreased compared with control, implying that the infectious pneumonia in vitro model was successfully constructed by exposing BEAS-2B cells to LPS. However, the decrease induced by LPS could be partially recovered after treated with 25-OH-VD (*p* < 0.01). Next, the expression levels of upstream proteins of the TGFβ/YAP/TAZ pathways (including CYP27B1 and VDR) before and after 25-OH-VD treatment were analyzed. The results are shown in Figure 3b. The mRNA expression levels of CYP27B1 and VDR in both the control and LPS groups were very low (*p* > 0.05), whereas the presence of 25-OH-VD significantly up-regulated the expression levels of both CYP27B1 and VDR. However, LPS treatment significantly enhanced TGFβ expression as compared with the control group, and VD treatment partially counteracted the enhancement in TGFβ induced by LPS administration. Then, the positive expression of nuclear translocated YAP in each group was detected using ICC. As expected, the increasing trend in LPS group was significantly reversed after 25-OH-VD treatment (Figure 3c). Similarly, the nuclear translocation of YAP/TAZ complex was evaluated using IFA. The results shown in Figure 3d suggest that the nuclear translocation rate of YAP/TAZ complex was notably increased after treatment with LPS. It then reduced after co-treatment with 25-OH-VD.

Finally, the changes in pro-inflammatory indicators were tested using the Western blot method. Importantly, the expression levels of IL-2, IFN-γ, TNF-α, and IL-1β were substantially up-regulated in the LPS group and decreased after treatment with 25-OH-VD (Figure 3e, *p* < 0.05). Thus, 25-OH-VD promoted the mRNA expression of CYP27B1 and VDR. However, it inhibited the nuclear translocation of YAP/TAZ complex to accelerate degradation, and thus decreased the levels of the inflammatory indicators. These findings indicate that 25-OH-VD inhibited the nuclear translocation of YAP/TAZ complex in BEAS-2B cells induced by LPS.

## Discussion

CRP, PCT, and IL-6 have significant roles in the diagnosis of inflammatory diseases [39]. In 1994, Patel *et al*. reported CRP as the first acute phase reaction protein, as its serum level can increase sharply in response to infection or acute trauma [40]. In 2001, Yukioka *et al*. found that PCT is a highly sensitive and specific indicator for the diagnosis of sepsis [41]. Additionally, IL-6 is an indicator of systemic inflammatory response syndrome (SIRS) with a close association with the severity and mortality of SIRS [42]. In this study, we tested the correlation between 25-OH-VD and the inflammatory biomarkers Ca^2+^, PCT, CRP, and IL-6 in NIP and found that there was no correlation between the serum levels of Ca^2+^, CRP, and 25-OH-VD, while the serum levels of PCT and IL-6 were negatively correlated with that of 25-OH-VD.

Serum level of 25-OH-VD is an internationally recognized standard for the nutritional evaluation of vitamin D [43]. Li *et al*. concluded that serum level of 25-OH-VD was related to community-acquired pneumonia, and was negatively correlated with disease severity [44]. Several studies have shown that the level of 25-OH-VD in cord-blood was negatively correlated with the risk of respiratory infections and wheezing in children [45–47]. In our study, the serum levels of 25-OH-VD and VDR decreased with an increase in the severity of infectious pneumonia, which was consistent with previous reports.

**Figure 4.**
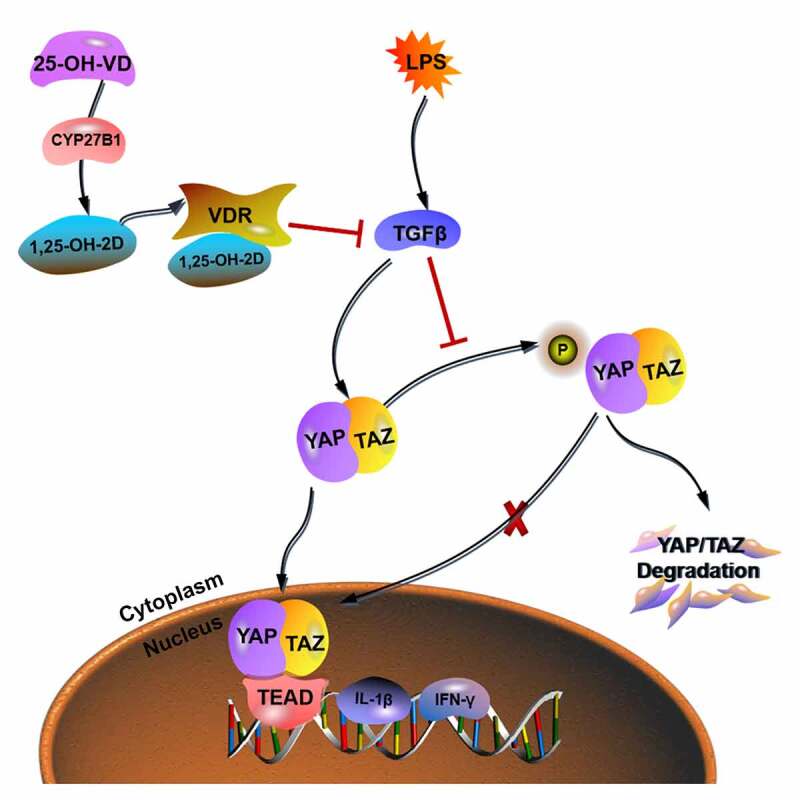
**The anti-inflammatory mechanism of 25-OH-VD in NIP**. When 25-OH-VD diffused into BEAS-2B cells, it was secondarily hydroxylated to generate 1,25-OH-2D under the action of CYP27B1 and combined with VDR to inhibit the activation and expression of TGFβ, thus reducing the nuclear translocation of YAP/TAZ complex. Once the YAP/TAZ complex was translocated into the nucleus, as the promoter, it participated in the expression levels of inflammatory factors (IL-2, TNF-α, IL-1β, and IFN-γ)

SARS-CoV-2 infectious pneumonia has been found attributable to vitamin D insufficiency and vitamin D supplementation may prevent infection to some extent [48]. The key role of YAP/TAZ in lung diseases is being gradually highlighted. For instance, the YAP/TAZ signaling dysregulation as one of the leading causes of pulmonary diseases including lung infection [49]. TAZ has been found to be involved in the pathophysiology of pulmonary diseases [49]. In the present study, 25-OH-VD was confirmed as an important up-stream molecule in the regulation of YAP/TAZ signaling. Not only can it directly up-regulate the expression level of VDR and decrease the nuclear translocation of YAP/TAZ complex, but it also reduces the expression of downstream pro-inflammatory indicators. These findings were consistent with those of previous studies. Therefore, as shown in Figure 4, the anti-inflammatory mechanism of 25-OH-VD is achieved via binding to VDR to reduce the expression of TGFβ and further block the nuclear translocation of YAP/TAZ complex, thereby inhibiting the secretion of downstream pro-inflammatory indicators.

## Conclusion

In summary, a low serum level of 25-OH-VD was negatively associated with a lower risk of NIP and a significant correlation between 25-OH-VD level and pneumonia indicators (PCT and IL-6) was detected. The present study explored the TGFβ/YAP/TAZ-dependent anti-inflammatory mechanism of 25-OH-VD and provided a novel approach for the prevention and diagnosis of vitamin D deficiency-induced NIP.

## Data Availability

The analyzed data sets generated during the present study are available from the corresponding author on reasonable request.

## Highlights

1. 25-OH-VD deficiency was a characteristic of severe pneumonia.
2. 25-OH-VD was negatively correlated with PCT and IL-6 in pneumonia.
3. YAP and TAZ were highly active in severe pneumonia.
